# Medical Assessment and Management of Self-Inflicted Head Injury in an Inpatient Child and Adolescent Mental Health Services (CAMHS) Setting

**DOI:** 10.1192/bjo.2022.474

**Published:** 2022-06-20

**Authors:** Georgie Patrick, John Smallwood, Cara Webb

**Affiliations:** Fairfield General Hospital, Manchester, United Kingdom

## Abstract

**Aims:**

To ascertain whether current medical assessment and management of self-inflicted head injuries in an inpatient CAMHS setting conforms with current NICE guidance.

**Methods:**

Incidents of self-inflicted head injury were identified on the incident logging system Ulysses. Incidents were matched to entries on Paris, the online clinical notes system. Data were collected from Paris on whether the incident was reviewed by a doctor, time until doctor review and which components of the NICE guidance were completed during the review. The data were collated into an Excel spreadsheet and analysed.

Inclusion criteria were CAMHS inpatients at 1 Greater Manchester hospital during November 2021 who had an incident of ‘head banging’ recorded on Ulysses. Exclusion criteria were patients on ward A as the ward was found to have its own care plans for managing head banging rather than escalating to doctors.

**Results:**

There were 52 incidents of head banging logged. 56% (n = 29) of incidents received a doctor review and 32% (n = 17) did not. For 10% (n = 5) of incidents a doctor review was declined and for 2% (n = 1) a review was conducted for another indication. The mean time taken until review was 4.3 hours with a range of 1 to 16 hours.

NICE guidance lists 9 components of the history that should be covered. 1 component met the 100% target and 1 component was documented in > 50% of incidents. The remaining 7 components were documented in < 50% of incidents.

NICE guidance lists 16 components of physical examination that should be completed. No components of the physical examination met the 100% target. 5 components were documented in > 50% of incidents. The remaining 11 components were documented in <50% of incidents.

NICE guidance recommends verbal and written safety netting advice is given. Advice was given in 16% (n = 5) of incidents. NICE recommends a responsible adult remains with the patient for 24 hours, this was documented in 77% (n = 22) of incidents. NICE recommends ongoing doctor concerns necessitate patient transfer to A&E. Concerns/lack of concerns were documented in 6.6% (n = 2) of incidents.

**Conclusion:**

This audit has demonstrated inconsistencies between doctor's documentation of self-inflicted head injuries in an inpatient CAMHS setting. The reviews do not meet the standards outlined by NICE. There is a good emphasis on gross neurology but less awareness of the need to document more subtle pathology and ongoing monitoring requirements.

